# Drug Coated Balloon in Young Patients With ST‐Elevation Myocardial Infarction: A Viable Treatment Option?

**DOI:** 10.1002/ccd.31699

**Published:** 2025-06-18

**Authors:** Davor Vukadinović, Saarraaken Kulenthiran, Felix Mahfoud, Bruno Scheller

**Affiliations:** ^1^ Innere Medizin III—Kardiologie, Angiologie und internistische Intensivmedizin Universitätsklinikum des Saarlandes Homburg Germany; ^2^ Department of Cardiology University Heart Center, University Hospital Basel Basel Switzerland; ^3^ Institute for Medical Engineering and Science Massachusetts Institute of Technology Cambridge Massachusetts USA; ^4^ Klinische und Experimentelle Interventionelle Kardiologie Universität des Saarlandes Homburg Germany

## Abstract

This clinical case highlights the safety and feasibility of drug coated balloon (DCB) angioplasty in a very young patients presenting with STEMI (ST‐Elevation Myocardial Infarction) to avoid long‐term complications associated with stent implantation, which still amount to 1%−2%. We successfully treated a 25 years old patient with occluded right coronary artery (RCA) with DCB after careful lesion preparation. Moreover, in a control angiogram 7 weeks later the RCA was patent with substantial lumen enlargement of 0.4 mm (RAO projection) to almost 2 mm (LAO projection) in a very short period of time. DCB angioplasty offers a viable alternative to stent implantation in young patients, avoiding the complications associated with permanent metallic implants while promoting vascular remodeling. While we acknowledge that more evidence is needed to optimize patient selection and confirm the long‐term benefits of DCB in ACS, this case highlights its efficacy and safety in terms of an individualized treatment strategy.

## 1 Introduction

The concept of drug coated balloon (DCB) was initially developed to address restenosis associated with implanted intracoronary stents. Additionally, it offers advantages such as the absence of a permanent metallic implant and improved vasomotion. Based on solid level of evidence it has been widely accepted across scientific community for treatment of in‐stent restenosis [1, 2, 3, 4] and small vessel disease [5]. Although there is evidence of non‐inferiority compared with drug eluting stents (DES) in patients with acute coronary syndrome (ACS) [6, 7, 8] DES are preferred and recommended treatment strategy in patients with ACS from European Society of Cardiology (ESC Guidelines) [9].

## 2 Case Details

**Figure 1:**
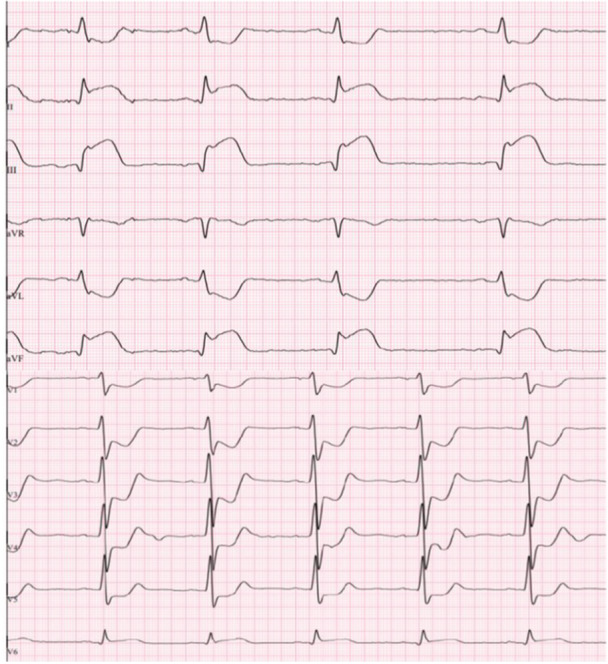
Electrocardiogram at presentation depicting ST‐elevation in leads II, III, aVF accompanied with ST‐depression in V1−V5 suggesting occluded vessel that supplies inferior wall. [Color figure can be viewed at wileyonlinelibrary.com]

A 25‐year‐old male presented to the emergency department (ED) with chest pain and discomfort. His blood pressure was markedly elevated at 180/90 mmHg. A 12‐lead ECG indicated acute ST‐elevation myocardial infarction (STEMI) with significant ST‐elevations (up to 0.4−0.5 mV in III and aVF) in inferior leads and reciprocal ST‐depressions in I, aVL, V1−V5 (Figure 1). The patient received 5.000 IE of heparin and 250 mg of aspirin intravenously. He was hemodynamically stable and transported to the cath‐lab for emergent coronary angiogram within 15 min. The patient had no prior history of chronic illness or regular medication use but was exposed to several cardiovascular risk factors, including cigarette smoking, arterial hypertension, obesity (body mass index of 37.2 kg/m^2^), and hyperlipidemia (LDL 125 mg/dL, normal < 116 mg/dL).

**Figure 2:**
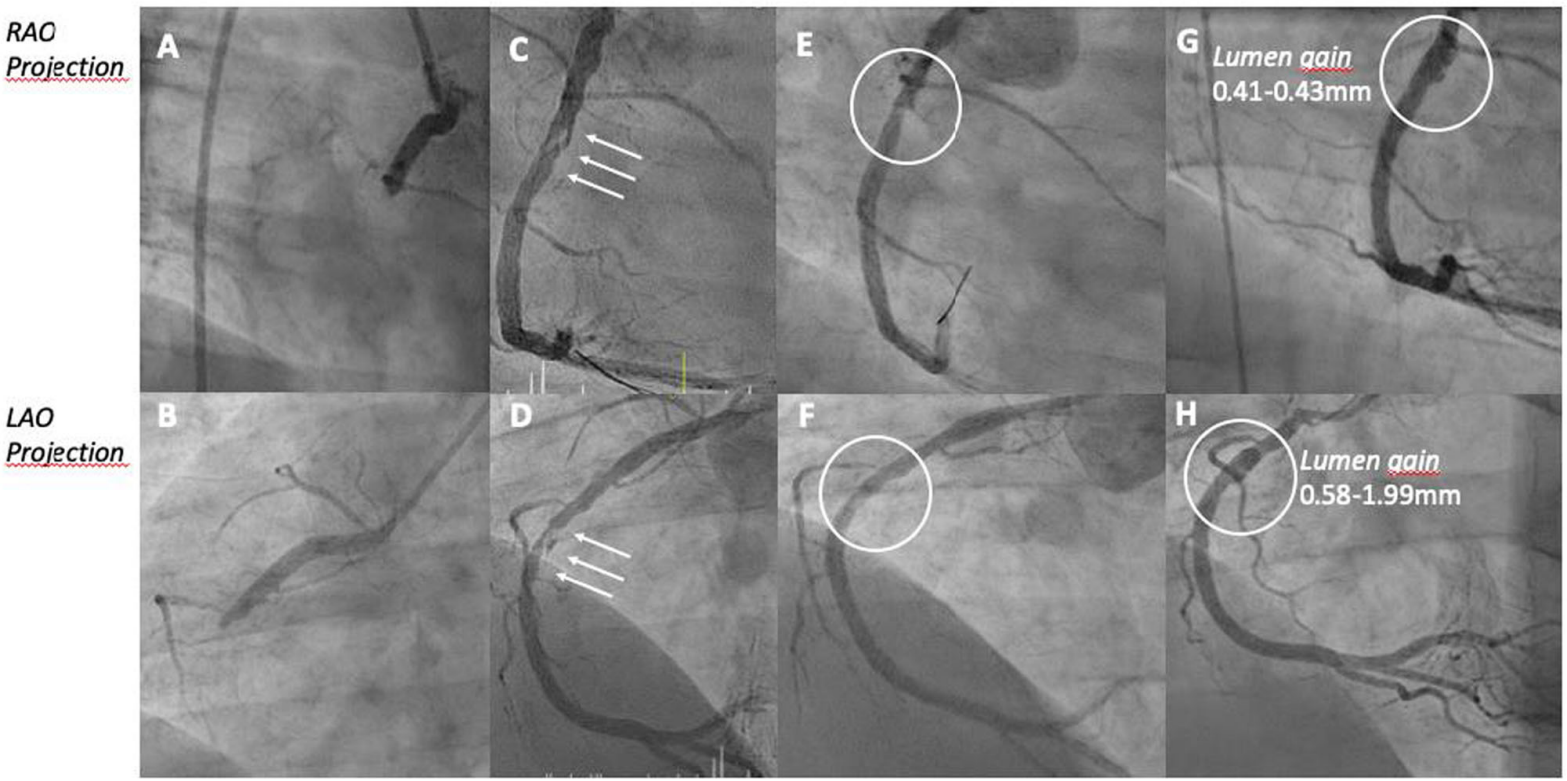
Coronary angiogram depicting occluded right coronary artery at index presentation (RAO projection—A; LAO projection—B); patent RCA after PTCA with semi‐compliant balloon and Scoring‐balloon (RAO projection—C; LAO projection—D), please note dissection membrane (white arrows); patent RCA after treatment with DCB and initial PCI in RAO (E) and LAO (F) projection; patent RCA after 7 weeks with lumen gain of 0.41−0.43 mm in RAO projection (G) and of 0.58−1.99 mm in LAO projection (H). [Color figure can be viewed at wileyonlinelibrary.com]

Coronary angiogram revealed occluded proximal right coronary artery (RCA) (Figure 2A,B). The left coronary system showed signs of advanced atherosclerosis without stenoses (sign of angiographic narrowing > 50% of the vessel diameter). During coronary angiography, the patient received loading dose (60 mg) of prasugrel and additional heparin. Percutaneous revascularization was performed. Following wiring of the culprit lesion, an over‐the‐wire balloon confirmed true intracoronary lumen position by contrast media injection. This was followed by lesion preparation using a semi‐compliant balloon with size of 2.5 × 15 mm (Sequent Neo, B.Braun) inflated with 8 ATM. Primary result showed a significant lumen gain with small degree of residual stenosis (20%) accompanied with no flow‐limiting type‐B dissection (Figure 2C,D). Furthermore, a prompt TIMI III was established. Plaque‐modification was further performed with a scoring‐balloon (size of 3.5 × 13 mm, NSE Alpha Lacrosse; inflation with 6 ATM). The final treatment of the culprit lesion involved a paclitaxel coated balloon (size 4.0 × 20 mm, Prevail Medtronic; inflation with 8 ATM), achieving optimal results with no significant residual stenosis, regression of dissection to type‐A, and prompt TIMI III flow (Figure 2E,F). The further clinical course was without complications. Laboratory tests revealed increased cardiac biomarkers, including peak high‐sensitive Troponin T of 5348 pg/ml (normal range: 0−14 pg/mL) and creatinine kinase of 3647 U/L (normal range: up < 190 U/L). Echocardiography showed mildly reduced ejection fraction with hypokinetic inferior wall but no valvular dysfunction. Given the patient's age, additional testing excluded rare hereditary causes that promote arterial thrombosis like thrombophilia, anti‐phospholipid syndrome and JAK2 gene mutation. Lipoprotein (a) was with 26.9 nmol/L within normal range (< 75).

Patient recovered fully and was discharged 1 week after admission. A follow‐up coronary angiogram 7 weeks later revealed an excellent mid‐term outcome, with no residual stenosis or dissection in the treated segment of the RCA (Figure 2G,H). Moreover, according to the quantitative coronary angiography (QCA) analysis a lumen enlargement of about 0.41−0.43 mm in right anterior oblique (RAO) and from 0.58 to 1.99 mm in left anterior oblique (LAO) projection, consistent with late lumen enlargement after DCB treatment have been documented.

## 3 Discussion

This clinical case highlights the safety and feasibility of DCB angioplasty in a very young patients presenting with STEMI thereby avoiding long‐term complications associated with stent implantation [10].

Paclitaxel, the antiproliferative agent used in our DCB, has been shown to be effective in porcine models [11]. Its clinical efficacy has been confirmed in proof‐of‐concept clinical trial several years later [12]. Several randomized clinical trials (RCT) have established the efficacy and safety of DCB treatment in a various clinical settings, in‐stent‐restenosis [1, 2, 3, 4], including small vessel disease [5, 13], non‐STEMI (NSTEMI) [6] and STEMI [7, 8]. The BASKET SMALL 2 trial demonstrated non‐inferiority of DCBs compared to drug‐eluting stents in reducing cardiac death and myocardial infarction in ACS patients. After 3 years of follow‐up the rates of major adverse events were similar without significant interaction between DCB and DES group [14].

In most RCTs, DCB showed non‐inferiority over DES used in these trials. In addition, according to a retrospective analysis of 1139 patients with STEMI due de novo disease treatment with DCB was comparable to a treatment with DES in a full or a propensity‐matched cohort of patients in term of all‐cause mortality and net adverse cardiac events (like CV mortality, ACS, ischemic stroke or transient ischemic attack, major bleeding and unplanned target lesion revascularization [TLR]) [15]. Additionally, analyses from the Swedish SCAAR registry in a propensity matched population (almost 2400 patients) have shown comparable outcomes between DCB and DES in terms of TLR, with a lower risk of target lesion thrombosis (TLT) for DCB [16].

While DES implantation remains the standard of care in ACS per current guidelines from the ESC (Level of recommendation 1, class of evidence A) [9], this case report supports the consideration of DCB‐only angioplasty in selected patients, particularly young individuals, to minimize long‐term complications associated with stents. Late lumen enlargement observed in this case within 7 weeks highlights the rapid atheromatous regression and vessel remodeling associated with paclitaxel‐coated DCBs.

The recently published REC‐CAGERFREE I trial compared DCB and DES treatment in non‐complex coronary lesions at 43 sites in China. At 2 years, cardiovascular death, target vessel myocardial infarction and TLR occurred in 6.4% in the DCB and 3.4% in the DES groups, not meeting the margin for non‐inferiority. The event rates at 2 years were extraordinarily low in both groups. Nevertheless, the study shows the safety of DCB‐only angioplasty: the rate of acute occlusions (0 vs. 1) and vessel thrombosis (0.4% vs. 0.3%) were very low in both groups. Interestingly, in the more complex subgroups of small vessel and bifurcation lesions the advantage of the DES disappeared [17]. In the large vessel cohort, an acceleration of events occurred in the DCB group, contrary to the characteristic flattening of the event curve beyond the first year seen in all trials with the original paclitaxel‐iopromide or paclitaxel‐urea DCB's [18, 19, 20] which may be explained through the unusual spray‐coating applied here.

The rate of stent‐related complications after 1 year remains around 2%−3% [10, 21] being even higher with increased number and length of the stents [10]. The basic idea and main advantage of DCB‐only strategy is absence of the permanent metal cage within the vessel as after stent implantation. This results in maintenance of vasomotion after treatment with DCB in comparison with stents [22]. Late lumen enlargement is another very valuable feature that is present in more than two‐thirds of patients after DCB treatment [23]. The presumed mechanism for this is the regression of atherosclerosis in the segment treated with paclitaxel [24]. In line with these findings in our case we could also detect extensive lumen gain in very short period of time (only 7 weeks), that indicates that this process occurs rapidly after treatment. However, it remains debatable the role of antiplatelet therapy in increasing the lumen due to the breakdown of remaining thrombotic material. Use of intracoronary imaging (IVUS or OCT) would provide more valuable insights concerning this issue. Unfortunately, intracoronary imaging was not performed in this patient. However, the current consensus among experts is that only angiographic criteria are decisive for the success of DCB treatment. Extensive lumen enlargement soon after DCB treatment may raise concerns that this treatment may facilitate the occurrence of coronary artery (CCA) aneurysms. According to a comprehensive analysis addressing this issue of 380 PCIs with paclitaxel‐coated DCB, there was no evidence of an unexpectedly high rate of CCA [25]. The rate was 0.8%. Moreover, according to the results of a meta‐analysis including 4,590 patients from 26 RCTs, PCI treatment with DCB (with paclitaxel) was associated with lower all‐cause and cardiac mortality compared with the control group (PCI with BMS, DES, POBA) at 1 and 3 years [26].

## 4 Conclusions

This case highlights successful treatment of a 25‐year‐old STEMI patient with DCB angioplasty, demonstrating extensive lumen gain and favorable short‐term outcomes. DCB angioplasty offers a viable alternative to stent implantation in young patients, avoiding the complications associated with permanent metallic implants while promoting vascular remodeling. Further studies are warranted to optimize patient selection and confirm the long‐term benefits of DCBs in ACS.

## Conflicts of Interest

F.M. is supported by Deutsche Gesellschaft für Kardiologie (DGK), Deutsche Forschungsgemeinschaft (SFB TRR219, Project‐ID 322900939), and Deutsche Herzstiftung. Saarland University has received scientific support from Ablative Solutions, Medtronic and ReCor Medical. F.M. has received speaker honoraria/consulting fees from Ablative Solutions, Amgen, Astra‐Zeneca, Bayer, Boehringer Ingelheim, Inari, Medtronic, Merck, ReCor Medical, Servier, and Terumo. B.S. reports payments or honoraria from Medtronic and B.Braun; and stock options with InnoRa GmbH. The other authors declare no conflicts of interest.
